# Immediate post-operative pain in anterior cruciate ligament reconstruction surgery with bone patellar tendon bone graft versus hamstring graft

**DOI:** 10.1186/s13018-016-0399-5

**Published:** 2016-06-08

**Authors:** Ravi Gupta, Dheeraj Kapoor, Love Kapoor, Anubhav Malhotra, Gladson David Masih, Anil Kapoor, Shweta Joshi

**Affiliations:** Department of Orthopaedics, Government Medical College and Hospital, Chandigarh, India; Department of Anaesthesia, Government Medical College and Hospital, Chandigarh, India

**Keywords:** Pain score, Immediate post-operative, BPTB graft, Hamstring graft, ACL surgery

## Abstract

**Background:**

Pain in the immediate post-operative period after anterior cruciate ligament (ACL) surgery, apart from an unpleasant experience for the patient, can act as a barrier for static quadriceps contractions and optimum execution of the initial rehabilitation protocol resulting in slow recovery and a later return to full function for a sportsperson. There is no report in the literature comparing pain in the immediate post-operative period after using the two most widely used autografts, bone patellar tendon bone (BPTB) graft and hamstring graft.

**Methods:**

The present study compared the visual analogue scale (VAS) pain score in the immediate post-operative period after arthroscopic ACL reconstruction with the BPTB and hamstring autografts. Both groups consisted of 50 patients each. The mean age of the BPTB and hamstring cohorts was 26.9 ± 7.3 years (age range 18–59 years) and 26.7 ± 9.0 years (age range 17–52 years), respectively. Unpaired *t* test was applied to compare pain scores between the BPTB and hamstring cohorts.

**Results:**

In the present study, patients in the BPTB cohort showed higher mean pain scores across all the post-operative time intervals except at 6 h. However, the difference in the mean VAS pain score at post-operative 6, 12,18, 24, 36 and 48 h in the two groups was statistically not significant (*p* value of 1, 0.665, 0.798, 0.377, 0.651 and 0.215 at 6, 12, 18, 24, 36 and 48 h, respectively).

**Conclusions:**

Our study concludes that the arthroscopic ACL reconstruction with BPTB autograft and hamstring autograft is associated with similar pain in the immediate post-operative period. As a result, aggressive physiotherapy regime is not affected by the type of graft being used for ACL reconstruction, as the pain scores in the immediate post-operative period are similar for both techniques.

**Trial registration:**

Clinical Trials Registry-India, CTRI/2016/01/006502

## Background

Pain in the immediate post-operative period, due to surgical trauma, can be an important barrier for starting an early rehabilitation programme after anterior cruciate ligament (ACL) reconstruction surgery, which can in turn delay the restoration of quadriceps contractions and range of motion at the knee joint. Additionally, the post-operative pain can result in an unpleasant experience of surgery for the patient. Whether there is any difference between the two most commonly used grafts for ACL reconstruction, the BPTB and hamstring grafts, in terms of pain in the immediate post-operative period, has not been reported in the international literature so far.

The relative merits of these two autograft types have been extensively investigated in the long term. The BPTB autograft has the reported advantage over the semitendinosus-gracilis (STG) autograft of having bone plugs on each end of the graft that provide excellent fixation points for the graft-screw interface and rapid healing within bone tunnels [1–7]. However, the BPTB graft is associated with more morbidities than the STG graft such as anterior knee pain, kneeling pain and higher chances of osteoarthritis of the knees, risk of patellar fracture and patellar tendon weakness or rupture [1, 8–12].

Thus, there is enough data in the literature comparing the results of ACL reconstruction using the BPTB and STG autografts. However, there is still no consensus regarding the superiority of one graft over the other [13, 14].

The present study was designed to compare pain in the immediate post-operative period after arthroscopic ACL reconstruction by the same surgeon using hamstring tendon autograft and patellar tendon autograft while following a similar aggressive post-operative rehabilitation protocol in both groups. In this study, we hypothesized that the use of the STG autograft in ACL reconstruction is associated with less pain in the immediate post-operative period as compared to the BPTB autograft.

## Methods

After approval by the Government Medical College and Hospital ethics committee (Chandigarh) and obtaining written informed consent from all the patients, 100 male patients presenting with ACL tear were enrolled in the study. The diagnosis of ACL tear and associated injuries was based on clinical examination (Lachman test, anterior drawer test and pivot shift test) and magnetic resonance imaging (MRI) of knee joint. Out of 100 patients, 50 patients underwent arthroscopic ACL reconstruction surgery using BPTB graft [15] and 50 patients underwent arthroscopic ACL reconstruction surgery using STG graft [16]. Patients were excluded, if they had previously been operated upon the same knee, with previous infective pathology in the same knee, an associated injury to posterior cruciate ligament or any other ligaments in the same knee, patients with any history of psychiatric illness or receiving any medications for such illness, patients on analgesics for any other ailment, patella fracture during graft harvesting, skeletally immature patients and patients operated under anaesthesia other than spinal anaesthesia.

The study design was prospective, randomized and controlled. Using a computer-generated random number table, patients were randomly allocated to either group A (*n* = 50) or group B (*n* = 50). Allocation concealment was done using sequentially numbered coded sealed envelopes. In group A, ACL reconstruction was done by using BPTB graft, in which the size of the bone plug was 10 mm in all the patients. In group B, ACL reconstruction was done by using quadrupled STG graft.

**Fig. 1:**
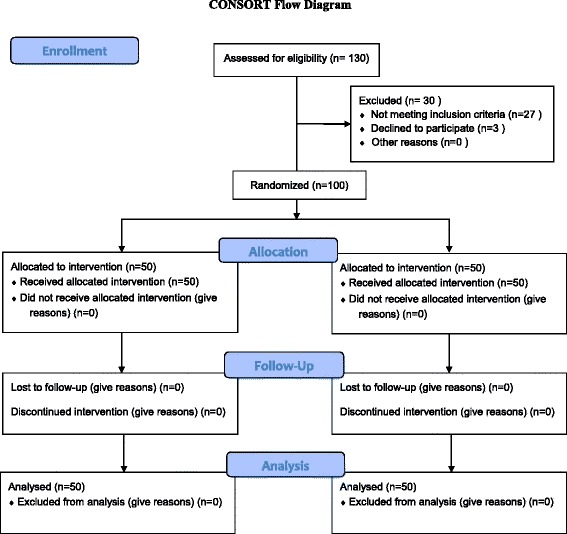
CONSORT flow diagram

All the patients were administered subarachnoid block using 2 ml of 0.5 % *w*/*v* bupivacaine in 8 % dextrose (hyperbaric solution) by an anaesthesiologist having more than 5-year experience in anaesthesia practice. No intravenous analgesics were given in the intraoperative period. The surgical and anaesthetic technique, post-operative rehabilitation and pain management were standardized in all the patients. The CONSORT flow diagram for the study is given in Fig. 1.

### Post-operative management

After surgery, both groups underwent the same rehabilitation protocol. A long knee brace was applied to all patients. Isometric quadriceps exercises, knee bending and active straight leg raising were started on day 0 as per tolerance of pain by the patient immediately after surgery when the motor block of spinal anaesthesia had regressed and patients were encouraged to bear the full weight depending upon pain tolerance. Patients were advised to walk with a brace locked in full extension. In case any patient experienced severe pain, injection of tramadol hydrochloride (100 mg) by intravenous route was administered as rescue analgesia.

### Outcome evaluation

In each patient, a generic unidimensional pain questionnaire with visual analogue scale (VAS) scores was recorded, starting at 6 h post-surgery (when the effect of the spinal anaesthesia had regressed), every 6 h for the first 24 h post-surgery and then every 12 h till 48 h post-surgery. The VAS used was a continuous scale comprising of a horizontal line, 10 cm (100 mm) in length, anchored by two verbal descriptors, one for each symptom extreme. For pain intensity, the scale was anchored by “no pain” (score of 0) and “pain as bad as it could be” or “worst imaginable pain” (score of 10) on a 10-cm scale [17].

### Statistical analysis

We performed unpaired *t* tests to compare pain scores between the BPTB and STG cohorts. All tests of significance were two-sided, and the results were considered to be significant at *p* < 0.05. For pain scores, we calculated 95 % confidence intervals (CIs) of the difference of the means.

## Results

### Participants

The mean age of the BPTB cohort was 26.9 ± 7.3 years (age range 18–59 years). The mean age of the STG cohort was 26.7 ± 9.0 years (age range 17–52 years). Both groups were statistically comparable with respect to age (*p* value = 0.146). All participants of the study were male patients.

### Duration of surgery

The mean duration of surgery for the BPTB cohort was 55.46 ± 0.93 min (range 40–60 min). The mean duration of surgery for the STG cohort was 40.66 ± 0.64 min (range 30–50 min). The difference in the mean duration of surgery for the two groups was found to be statistically significant (*p* value = 0.0131).

### BPTB versus STG: VAS scores for pain

**Fig. 2:**
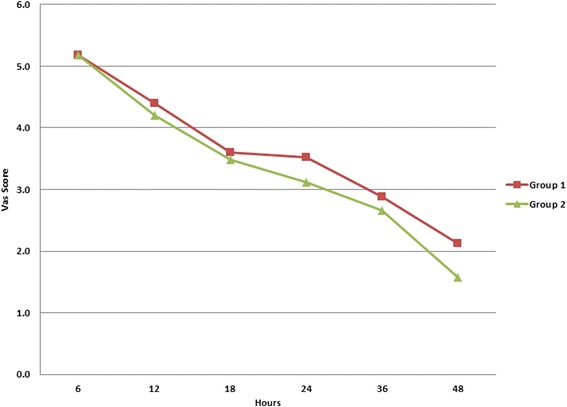
Graph showing comparison of the mean VAS pain score for the two groups at specified intervals of time

Patients in the BPTB cohort showed higher mean pain scores across all the post-operative time intervals except at 6 h. However, the difference in the mean VAS pain score at post-operative 6, 12,18, 24, 36 and 48 h in the two groups was statistically not significant (*p* value of 1, 0.665, 0.798, 0.377, 0.651 and 0.215 at 6, 12, 18, 24, 36 and 48 h, respectively). The details of the mean VAS scores for the two groups over the 48-h post-operative period are as shown in Fig. 2 and Table 1.

**Table 1:** BPTB vs STG mean VAS pain score (in centimetres) at specified intervals of time

	Mean ± SD VAS score			
Post-operative time (h)	Group A (*n* = 50)	Group B (*n* = 50)	Mean difference	*p* value
	BPTB	STG	(95 % CI)	
6	5.2 ± 2.5	5.2 ± 2.4	0 (−0.97 to 0.97)	1
12	4.4 ± 2.3	4.2 ± 2.3	0.2 (−0.71 to 1.11)	0.665
18	3.6 ± 1.9	3.5 ± 2.0	0.1 (−0.67 to 0.87)	0.798
24	3.5 ± 2.4	3.1 ± 2.1	0.4 (−0.49 to 1.29)	0.377
36	2.9 ± 2.3	2.7 ± 2.1	0.2 (−0.67 to 1.07)	0.651
48	2.1 ± 2.1	1.6 ± 1.9	0.5 (−0.29 to 1.29)	0.215

## Discussion

The mean duration of surgery in the BPTB group was statistically higher as compared to the STG group. Further, harvesting of the BPTB graft involves the cutting of bone and periosteum on the patellar as well as tibial sides, thereby increasing the severity of surgical trauma in comparison to the STG graft. The longer duration of surgery and more surgical trauma in BPTB graft patients can be a cause of more pain in these patients as compared to STG patients. In the present study, patients in the BPTB cohort showed higher mean pain scores across all the post-operative time intervals except at 6 h (Table 1). We feel that the pain score at 6 h may have some confounding due to a residual analgesic effect of the spinal anaesthesia [18]. However, the difference in pain scores, even at other time intervals, in our study could not reach a statistically significant level.

The success of ACL surgery depends upon post-operative rigorous rehabilitation [19]. The role of surgical procedure is only to re-establish the physical structure of the ligament, whereas an early rehabilitation helps to maintain the physical and psychological capabilities of the athlete [20]. We, at our institution, follow an accelerated rehabilitation programme which has been shown to be safe and effective by various studies [19, 20]. According to this programme, patients are supposed to begin immediate post-operative weight bearing, move the knee from 0° to 90° of flexion and perform closed-chain strengthening exercises. Thus, a surgical procedure, which can provide less pain in the immediate post-operative period, will be more useful for the accelerated rehabilitation programme.

The decision regarding the choice of graft is currently an important point of debate in the treatment of ACL injury [13]. Although the long-term advantages and disadvantages of the BPTB and STG grafts are well known, there was no evidence in the literature as to which of the two grafts would provide less pain in the immediate post-operative period.

The residual analgesic effect of the central neuraxial block in the first few hours of the post-operative period may act as one of the confounding factors while appraising the pain intensity by the type of surgical procedure. Hence, an extended period of evaluation was done in the present study [18]. Various clinical trials have evaluated pain scores during the immediate post-operative period, with durations varying from 8 to 96 h [21–28]. In the present study, we evaluated pain intensity for the immediate 48-h post-operative period, because all the patients start full weight bearing and knee bending beyond 90° of flexion within the first 48 h as per the accelerated rehabilitation protocol followed by us.

McDonald et al. conducted a study in which a comparison of pain scores and opioid/analgesic medication use in the post-operative period, in patients undergoing single-bundle and double-bundle ACL reconstruction, was done. The study concluded that there was no difference in pain scores in the two groups; however, opioid use/analgesic medication use was more in the double-bundle group. Further, the patients who underwent surgery under spinal anaesthesia experienced less pain in the post-operative period than those who received general anaesthesia, as evidenced by significant difference in the consumption of analgesics amongst the two groups [29]. We operated on all the patients in both groups under spinal anaesthesia to remove this bias and its effects on pain scores in the post-operative period.

For the study, the major limitation is the use of tramadol hydrochloride to relieve pain. It was administered in a total of 12 patients amongst the two groups in the first 6 h only (7 and 5 in the BPTB and STG groups, respectively), and the use was comparable (*p* < 0.05). However, additional data in terms of the total dose of consumption and the time to first dose of rescue analgesia should be recorded to make the data more credible.

Since in our study the difference in pain scores could not reach a statistically significant level at any time interval, our hypothesis that the use of hamstring tendon autograft in ACL reconstruction is associated with less pain in the immediate post-operative period as compared to bone patellar tendon bone autograft could not be proved.

## Conclusions

Aggressive physiotherapy regime is not affected by the type of graft (BPTB or STG) being used for ACL reconstruction, as the pain scores in the immediate post-operative period are similar for both the techniques.

## Abbreviations

ACL, anterior cruciate ligament; BPTB, bone patellar tendon bone; CI, confidence interval; MRI, magnetic resonance imaging; STG, semitendinosus-gracilis; VAS, visual analogue scale.
